# Development of a Performance Measurement Framework for European Health Technology Assessment: Stakeholder-Centric Key Performance Indicators Identified in a Delphi Approach by the European Access Academy

**DOI:** 10.3390/jmahp14010005

**Published:** 2026-01-15

**Authors:** Elaine Julian, Nicolas S. H. Xander, Konstantina Boumaki, Maria João Garcia, Evelina Jahimovica, Joséphine Mosset-Keane, Monica Hildegard Otto, Mira Pavlovic, Giovanna Scroccaro, Valentina Strammiello, Renato Bernardini, Stefano Capri, Ruben Casado-Arroyo, Thomas Desmet, Walter Van Dyck, Frank-Ulrich Fricke, Fabrizio Gianfrate, Oriol Solà-Morales, Jürgen Wasem, Bernhard J. Wörmann, Jörg Ruof

**Affiliations:** 1 Secretariat of the European Access Academy (EAA), 4059 Basel, Switzerland; 2 Erasmus Centre for Health Economics Rotterdam, Erasmus University Rotterdam, 3062 PA Rotterdam, The Netherlands; 3 Erasmus School of Health Policy and Management (ESHPM), Erasmus University Rotterdam, 3062 PA Rotterdam, The Netherlands; 4 Global Heart Hub, H91 FF68 Galway, Ireland; 5 F. Hoffmann-La Roche Ltd., 4070 Basel, Switzerland; 6 Cancer Patients Europe (CPE), 1000 Brussels, Belgium; 7 SDA Bocconi School of Management, Bocconi University, 20136 Milan, Italy; 8 Sabouraud Research and Treatment Center for Scalp and Skin Diseases, Saint Louis Hospital, 75010 Paris, France; 9 Medicines Development and Training (MDT) Service, 75020 Paris, France; 10 Direzione Farmaceutico, Protesica, Dispositivi Medici. Area Sanità e Sociale, Regione Veneto, 30123 Venezia, Italy; 11 European Patients’ Forum (EPF), 1040 Etterbeek, Belgium; 12 School of Medicine, University of Catania, 95124 Catania, Italy; 13 School of Economics and Management, Cattaneo-LIUC University, 21053 Castellanza, Italy; 14 Cardiac Electrophysiology Laboratory, Université Libre de Bruxelles-Erasme Hospital, 1070 Brussels, Belgium; 15 European Society of Cardiology (ESC), 06903 Sophia Antipolis, France; 16 Healthcare Management Center, Vlerick Business School, 1210 Brussels, Belgium; 17 Department of Pharmaceutical and Pharmacological Sciences, KU Leuven, 3000 Leuven, Belgium; 18 Technische Hochschule Nürnberg, 90489 Nürnberg, Germany; 19 Department of Health Economics, University of Ferrara, 44121 Ferrara, Italy; 20 HiTT Foundation, 08015 Barcelona, Spain; 21 Institute for Health Care Management and Research, University of Duisburg-Essen, 45127 Essen, Germany; 22 German Association of Hematology and Oncology (DGHO), 10117 Berlin, Germany; 23 Division of Hematology, Oncology and Tumor Immunology, Department of Medicine, Charité-Universitätsmedizin Berlin, 10117 Berlin, Germany; 24 Hannover Medical School, Institute for Epidemiology, Social Medicine and Health System Research, 30625 Hannover, Germany

**Keywords:** EUHTA, key performance indicators, PICO, health policy, health technology assessment, European access academy

## Abstract

Background: The objective of this work was to support the implementation of the European Health Technology Assessment Regulation (EU HTAR) and optimize performance of the evolving EU HTA system. Therefore, an inclusive multi-stakeholder framework of key performance indicators (KPI) for success measurement was developed. Methods: A modified Delphi-procedure was applied as follows: (1) development of a generic KPI pool at the Fall Convention 2024 of the European Access Academy (EAA); (2) review of initial pool and identification of additional KPIs; (3) development of prioritized KPIs covering patient, clinician, Health Technology Developer (HTD), and System/Member State (MS) perspectives, and (4) consolidation of the stakeholder-centric KPIs after EAA’s Spring Convention 2025. Results: Steps 1 and 2 of the Delphi procedure revealed 14 generic KPI domains. Steps 3 and 4 resulted in four prioritized KPIs for patients (patient input; utilization of patient-centric outcome measures; time to access; equity); six for clinicians (population/intervention/comparator/outcomes (PICO); addressing uncertainty; clinician involvement; transparency; equity and time to access); four for HTDs (PICO; joint scientific consultation (JSC) process; joint clinical assessment (JCA) process; time to national decision making); five from a system/MS perspective (PICO; learning and training the health system; reducing duplication; equity and time to access). The scope of, e.g., the PICO-related KPI, differed between stakeholder groups. Also, several KPIs intentionally reached beyond the remit of EU HTA as they are also dependent on MS-specific factors including national health systems and budgets. Discussion and Conclusions: The KPI framework developed here presents a step towards the generation of systematic multi-stakeholder evidence to support a successful implementation of the EU HTAR. The relevance of the identified stakeholder-centric KPIs is confirmed by their alignment with the Health System Goals suggested in the context of “Performance measurement for health improvement” by the World Health Organisation. Implementation of the framework, i.e., measurement of KPIs, is envisioned to provide evidence to inform the 2028 revision of the EU HTAR.

## 1. Introduction

The aim of the Health Technology Assessment (HTA) is clearly stated in recital 3 of the European Union (EU) Regulation 2021/2282 on HTA (EU HTAR) [1]: ‘HTA is able to contribute to the promotion of innovation, which offers the best outcomes for patients and society as a whole and is an important tool for ensuring proper application and use of health technologies.’ As such, one of the EU HTAR’s ambitions is ‘to ensure that joint clinical assessments could effectively facilitate market access and contribute to the timely availability of innovative health technologies for patients.’ [1]. Although there is broad consensus on that goal, a deeper reflection on the language reveals the enormous ambition behind that vision. Looking back into the history of the regulation indicates how difficult it is to advance the ‘European Health Union’ [2] in such an important topic. The history of EU HTA started more than two decades ago [3,4,5,6]. Then, in 2010, the first EU-funded Joint Action (JA1) was installed; JA2 and JA3 followed in 2012 and in 2017, respectively, and still the regulation was not in place [3]. Finally, in 2021, a service contract was signed with the EUnetHTA 21 consortium to continue the joint work beyond the end of EUnetHTA JA3 [7,8,9]. Interestingly, while literature exists on the outcomes of the Joint Actions and the respective working packages, systematic performance measurement is sparse. A report commissioned by the European Federation of Pharmaceutical Industries and Associations (EFPIA) indicates that there is progress over time but still major procedural shortcomings across all the pilot assessments [10,11].

In January 2022, the EU HTAR entered into force [1]. The preparation phase of the regulation ended in January 2025 and transitioned to the application phase. The European Commission’s implementation rolling plan contains the list of key activities to be carried out in preparation for the implementation of the EU HTAR and includes the respective procedural achievements [12]. In addition, a limited body of literature on general progress and outcomes is available [4,13,14]. However, there was no systematic assessment of successful preparation, and the rolling plan fails to evaluate whether the completed actions achieve their intended goals. Critical voices were raised indicating that, e.g., methodological guidances that were published by the coordination group do not match the principles of evidence-based medicine or the requirements of evolving targeted medicinal product development characteristics [15,16,17].

To fill this gap and prepare for a meaningful performance review of EU HTAR, the European Access Academy (EAA) aimed to develop a systematic framework of stakeholder-centric Key Performance Indicators (KPIs) to critically support and track the fulfilment of the ambition embedded in the regulation [1]. In line with the conceptual base of the assessment methods suggested within the regulation, we applied the ‘Population/Intervention/Comparator/Outcomes (PICO) Scheme’ when developing the scope of the measurement framework.

- The *Population* of interest includes individual patients as well as society as a whole (i.e., reflecting the perspective of all involved stakeholders and collaborators).
- The *Intervention* reflects the introduction of EU HTA.
- The *Comparator* is the plethora of previous national HTA processes across the Member States (MS).
- Finally, the *Outcomes* of interest included in this framework reflect the desired health system improvements.

Further, the overarching Health System Goals previously identified for performance measurement of health systems by The World Health Organisation (WHO) were referred to for evaluation of the KPI framework for EU HTA developed here [18,19].

The objective of this research is to support the implementation of the EU HTAR and eventually the improvement of EU health systems by developing an inclusive multi-stakeholder framework for measuring and optimizing performance of the envisioned learning and evolving EU HTA system.

## 2. Methods

To identify a prioritized, stakeholder-centric framework for measuring the success of EU HTA, a modified Delphi-procedure was applied including multi-stakeholder input in each step [20]. The initial generic KPI pool was based on survey responses at the EAA Fall Convention 2024 (step 1), review of initial KPI pool and identification of additional KPIs during the registration process for the EAA Spring Convention 2025, which resulted in a consolidated list (step 2). Discussions at the EAA Spring Convention 2025, based on the generic KPI pool from step 2, and taking into account additional input, e.g., keynote presentations and a review of performance criteria as embedded in the EU HTA Regulation, led to a list of prioritized stakeholder-centric KPIs (step 3). Further review and alignment of the identified KPIs by the authors yielded a final list of stakeholder-centric KPIs for performance measurement of EU HTA (step 4). In each step, input from multiple relevant stakeholder groups was collected. Subsequently, duplicates were removed and responses regarding the same or similar items were clustered as appropriate. Clustering was performed independently by a six-eye expert group (with multi-stakeholder and national backgrounds, including members of the EAA Secretariat) utilizing a keyword and context approach, resulting in a consensus pool. Where needed, consensus was reached by calling on additional experts from the EAA Faculty. The consensus pool was then reviewed and validated by the expert review panel before proceeding to the next step. The review panel included experts from several European countries (Belgium, France, Germany, Italy, Netherlands, Portugal, Serbia, Spain, Switzerland, and the United Kingdom) and various institutional backgrounds and expertise, including clinicians, patients’ representatives, regulators, HTA bodies, payers, academia, and Health Technology Developers (HTDs). In the last Delphi review, the expertise of all authors was included in discussions via written feedback and subsequent video calls.

**Figure 1:**
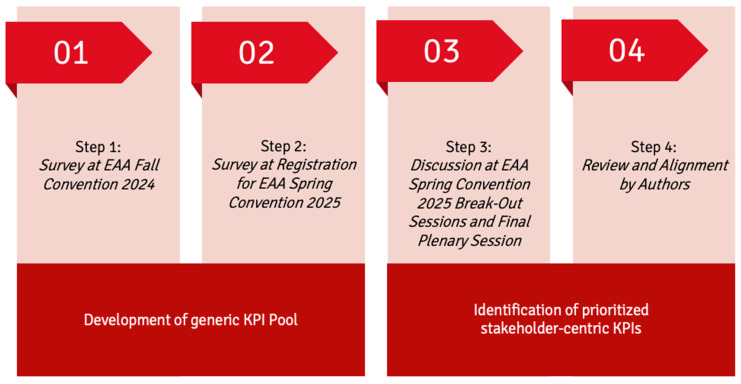
Modified Delphi approach for the identification of a stakeholder-centric KPI framework for EU HTA. Abbreviations: EAA: European Access Academy, EU: European Union, HTA: Health Technology Assessment, KPI: Key Performance Indicator.

An overview of the process flow is displayed in Figure 1.

### 2.1. Delphi Approach—Steps 1–2: Development of Generic KPI Pool

Step 1: At the EAA Fall Convention 2024, taking place on 24/25 October at LUISS Business School, Rome, Italy, participants were asked to “suggest one precise, measurable performance indicator for EU HTA”. To allow for both on-site and remote participants to respond, a Microsoft Forms Survey (Microsoft 365 by Microsoft Corporation, Redmond, WA 98052-6399, USA, online version as of 25 October 2024) was utilized.

Step 2: Registrations for the EAA Spring Convention 2025 were collected through a Microsoft Forms Survey which was distributed via various channels direct and indirect channels [21,22,23]. As part of the form, registrants were asked to provide details of any additional performance indicators they consider important. For data analysis, the responses were separated from the information regarding the registration for participation in the EAA Convention and the anonymized data was transferred into a Microsoft Excel (Microsoft^®^ Excel for Mac, Version 16.94) file for analysis. A preliminary analysis of responses was carried out for presentation at the EAA Spring Convention 2025 and for publication in the Convention Proceedings [24], the final data cut was 30 March 2025, leading to a new consolidated list of key performance indicators.

### 2.2. Delphi Approach—Steps 3–4: Identification of Prioritized Stakeholder-Centric KPIs

Step 3: The EAA Spring Convention 2025 took place on 3 April 2025, at the premises of the German Joint Federal Committee (Gemeinsamer Bundesausschuss, G-BA, Gutenbergstr. 13) in Berlin, Germany. It included plenary presentations in the morning session and parallel break-out sessions with five working groups (WG) in the afternoon. The convention was designed as a hybrid meeting, allowing for on-site and remote participation via public webstream and ZOOM. Four dedicated WG (WG 1–4) with approximately 15–20 allocated on-site participants each (exact numbers dependent on room capacities) plus one WG for all virtual participants were formed in advance. The allocation for WG 1–4 was based on specific participant characteristics: (i) personal and professional background; (ii) national diversity in each group; and (iii) stakeholder expertise and diversity within each group (clinicians’ representatives, patients and patients’ representatives, regulators, HTDs, HTA bodies, payers, policymakers, and academic representatives). The purpose of the five break-out sessions was to identify and prioritize three to five key performance indicators for EU HTA from a pre-specified perspective:

- WG 1: Patient perspective
- WG 2: Clinician perspective
- WG 3: HTD perspective
- WG 4: System-level/member state perspective
- WG 5: Birds’ eye view (i.e., integrative perspective)

In preparation of the break-out sessions, the keynote presenters were asked to include a slide with EU HTA performance measurement details from their specific perspectives and in a separate presentation before the break-out sessions, performance criteria embedded in the regulation were reviewed and discussed [1]. Prior to the convention, three co-leads were appointed for each WG by the EAA Secretariat, in consultation with the EAA Faculty based on stakeholder background and expertise. The co-leads were responsible for facilitating and structuring each respective session, as well as for encouraging active and balanced participation among all participants. To achieve consistency in approach and reporting between the WGs, the EAA secretariat and the leadership teams of the WGs had previously agreed upon the proposed structure and approach of the break-out sessions. Further, a predefined PowerPoint template (Microsoft^®^ Powerpoint for Mac, Version 16.94) provided by the EAA Secretariat was utilized by each group to capture discussion content and present outcomes in the final plenary session.

To support prioritization of predefined performance indicators as a starting point for discussions in the virtual WG 5, a ranking exercise was performed (using slido.com by Cisco Systems Inc., San Jose, CA, USA, online version as of 3 April 2025). The aggregated descriptive ranking results were displayed to the participants as the basis for further discussions in this group.

The results of the break-out sessions were presented in the final plenary session by each WG’s appointed representative and further discussed among all participants of the convention.

Step 4: A further review of the KPIs identified in the WGs after the convention, alignment and, where appropriate, minor amendments by the authors as per the procedure of consensus development described above, yielded the final list of stakeholder-centric KPIs.

## 3. Results

### 3.1. Delphi Approach—Steps 1–2: Development of Generic KPI Pool

The survey at the EAA Fall Convention 2024 yielded an initial KPI pool of ten distinct KPIs. The majority of KPIs cover procedural aspects such as timelines, system efficiency and capacities, MS participation, and Joint Clinical Assessment (JCA) report integration into MS systems. Others refer to content features, e.g., PICO, and several KPIs specify improvement in patient access.

This initial KPI pool was further refined and extended based on responses received during the registration for the EAA Spring Convention 2025. Overall, 180 responses were received and led to a consolidated list of 14 generic KPIs (see Table 1).

### 3.2. Delphi Approach—Steps 3–4: Identification of Prioritized Stakeholder-Centric KPIs

During the morning session of the EAA Convention, nine speakers presented their specific perspectives on success measurement for EU HTA and content discussions were initiated utilizing a roundtable format [25]. In addition, an overview on success criteria as embedded in the wording of the EU HTA Regulation was provided, including detailed legal references, and followed by in-depth discussions in smaller working groups [26,27].

The numbers of participants per break-out session were, respectively, 13 for patient perspective (WG 1), 14 for clinician perspective (WG 2), 21 for HTD perspective (WG 3), 22 for system-level/MS perspective (WG 4), and 28 for birds’ eye view (WG 5). Each break-out session included representatives of a variety of national backgrounds (Belgium, Croatia, France, Germany, Italy, Latvia, Lithuania, Netherlands, Poland, Portugal, Serbia, Spain, Sweden, Switzerland, and the UK), as well as stakeholder profiles (Supplmentary Material Figure S1).

Each group developed a list of key performance indicators for EU HTA from their specific perspective (WG 1: 4, WG 2: 3, WG 3: 4, WG 4: 3, and WG 5: 4) which were then presented and discussed in the final plenary session. Of the generic pool from steps 1 and 2, seven KPIs were prioritized by the WGs.

**Figure 2:**
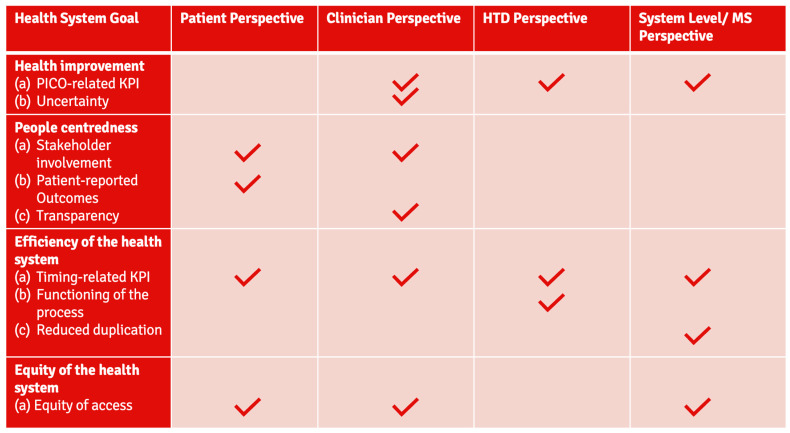
Alignment of the prioritized stakeholder-centric KPIs for EU HTA with the WHO’s health system goals [19]. Check marks represent prioritization of the respective health system goal by each stakeholder group. Abbreviations: EU: European Union, HTA: Health Technology Assessment, HTD: Health Technology Developer, KPI: Key Performance Indicator, MS: Member State, PICO: Population/Intervention/Comparator/Outcome, WHO: World Health Organisation.

The prioritized stakeholder-centric KPIs identified by the WGs at the convention were subsequently reviewed and aligned by the authors. As a result, a final list of KPIs was specified for each of the stakeholder perspectives (Patient: 4, Clinician: 6, HTD: 4 and System Level/MS: 5). There was substantial alignment between the stakeholder groups—the prioritized KPIs (see Table 2 and Figure 2) were as follows:

- Regarding timing: 4/4 groups,
- Regarding equity: 3/4 groups,
- PICO-related: 3/4 groups,
- Stakeholder involvement: 2/4 groups.

In addition, there were individual KPIs specified for single stakeholders only (see Table 2).

In detail, common KPIs were prioritized and discussed as follows:

KPIs regarding timing and equity of access:

Patient access, comprising both dimensions—equity of access, or availability, and time to access—was prioritized both from a patient’s and from a clinician’s point of view. Equity, i.e., availability in MS contingent on national budgetary constraints, and time to access, were also considered highly relevant from an MS/system-level perspective. Time to reimbursement was preferred as a metric over time to decision-making with respect to the goal of the regulation being to improve patient access. It was, however, acknowledged that these KPIs do not solely rely on the successful implementation of the EU HTAR as they would be influenced by other, MS-specific, factors including national health systems and budgets. In addition, medical technologies with negative reimbursement decisions would not be captured. Nevertheless, they were considered critical and necessary to include in any meaningful KPI framework for EU HTA. From the HTD perspective, an overall shorter duration of the decision-making process at the MS level was prioritized, which correlates with time to patient access.

PICO-related KPIs:

KPIs related to the PICO scheme were prioritized from a clinician, HTD, and MS/system-level perspective. While there was common ground on the key aspect in question with respect to the PICO, i.e., the comparator selection, the scope of the PICO KPI differed substantially for each of those stakeholders. From the clinician’s perspective, priority was given to the optimization of PICOs over time for a specific disease or indication, keeping in mind existing national treatment standards as well as evidence-based clinical standards. Similarly, a reduction in the number of PICOs was considered critical from a HTD’s point of view, not only to reflect a convergence of clinical standards but also to focus comparative evidence generation against the most relevant comparator(s) across the EU, and thus enable optimized clinical data development. Whilst the topic of PICOs was also prioritized from an MS/system-level point of view, this WG’s emphasized the need for exhaustiveness in the PICO to reflect the evidence-based clinical standards in the MS, as well as the fact that PICO discussions can play a key role in strengthening and “training” health systems.

KPIs on stakeholder involvement:

Successful or relevant and meaningful input, i.e., active involvement early and continuously, and transparency with regards to the contributions included and the related impact, was considered key for success of EU HTA both from a patient point of view and from the perspective of clinicians. Medical and scientific society input in all EU HTA processes covering the clinical development period, including Joint Scientific Consultations (JSC) and JCA, at EU and national level, were considered critical.

In detail, the following KPIs were prioritized from the perspective of single stakeholders:

From a patient perspective, the utilization of patient-centric/patient-relevant outcome measures as input describing real value for patients was prioritized. These measures should have an appropriate weight both in the assessment and in the subsequent decision-making process.

Furthermore, from the clinician perspective, fulfilment of transparency requirements and their balance with the inclusion of best available expertise within the process was considered critical for the successful implementation of EU HTA.

HTDs additionally prioritized a well-functioning JSC process which can address available demand and accommodate the dynamic nature of clinical development programs. JSCs are considered critical to enable HTDs to generate the appropriate evidence and present high-quality data in their EU HTA submissions. Workability and efficiency of the system with regards to the EU HTA processes, including realistic response timelines, and efficient and effective communication, were also prioritized by HTDs.

With a reduction in workload via streamlining of processes and decreased duplication of effort being a major goal of the EU HTAR, this was considered a high priority KPI from a system-level perspective, together with the utilization of the JCA reports in MS. While the latter is a given due to the wording of the regulation, where MS have to “give due consideration” to the report, the details of its implementation and effects on workload and system efficiency remain to be seen, and must be monitored.

## 4. Discussion

In 2008, the European Chapter of the WHO published the background document on ‘Performance measurement for health improvement’ [18]. In line with this guidance, the ultimate goal of the performance measurement framework presented in our study aims to promote the achievement of the health system objectives as set out in the EU HTAR and related documents [1]. The perspective chosen for this framework revolves—as suggested by both, the WHO and the European Observatory on Health Systems and Policies, and as has been stressed by many others in the field—around the real possibility for key stakeholders such as patients, clinicians, HTDs, and health system representatives, to contribute meaningfully to a successful implementation of the regulation [19,28,29,30]. While various developments since 2008—including technology advancements and a globalization of challenges, including in health—have had a profound and far-reaching impact on health systems, budgets, and payment frameworks, as well as definitions and approaches to performance measurement, key components of the framework laid out by Smith et al. [18] are still relevant, as discussed nearly 15 years later, in 2022, by Papanicolas et al. [19]. The prioritized stakeholder-centric KPIs are well aligned with the overall health system goals as identified by the WHO [19]: (i) health improvement, (ii) people centeredness, (iii) efficiency, and (iv) equity of the health system (see Figure 2).

KPIs covering the Health System Goal ‘health improvement’:

Addressing the PICO scheme closely relates to the health system goal ‘health improvement’ and was prioritized from a clinical, system-level, and HTD perspective. Ensuring exhaustiveness, i.e., an inclusive approach covering the joint requests of all member states is in line with recital 25 and article 2.9 of the EU HTAR with the minimum possible number of PICOs [1]. This is also consistent with recital 16 and article 9 of the Implementing Regulation on Joint Clinical Assessments (JCA-IR; Regulation (EU) 2024/1381), which highlight the required reduction in PICOs to the lowest possible number of parameter sets [31]. However, from a developer’s perspective, there is a need to focus and achieve convergence over time in order to meaningfully inform future clinical development programs [1,32,33]. Clinicians pointed to the necessity of PICO schemes being reflective of the clinical standards in the various countries while also suggesting a focus on the most effective comparative treatments. Further, there is a need to provide clarity to what extent available data address methodological uncertainties, in line with scientific evidence-based clinical decision making as per recital 5 of the EU HTAR [1]. While no PICO directly related to ‘health improvement‘ was prioritized from a patient perspective, improvement of patient health is clearly the ultimate goal of any health regulation. However, considering the striking inequity of care across Europe, it appears mandatory to prioritize equitable care as KPI and include related health improvement under that umbrella. The common denominator across all involved stakeholders was that the PICO schemes over time should support and inform the overarching health system goal of ‘health improvement’ of the population within the EU member states [19].

KPIs covering the Health System Goal ‘people centeredness’:

The role and importance of involvement of stakeholders, i.e., ‘people centeredness’ of the EU HTA processes, has been thoroughly studied and discussed in the field and was prioritized in both the patient and clinician perspective [14,28,29,34,35]. This is in line with recitals 20 and 44 of the regulation, which stress the importance of inclusive engagement and request involvement of external experts with in-depth specialized expertise in the JSC and JCA process [1]. Moreover, this corresponds with recital 13 and articles 8 and 9.1 of the JCA-IR. Article 8 allows for the elicitation of input from organizations related to patients and healthcare professionals, while article 9.1 of the JCA-IR concerns the input from patients, clinical experts, or other experts on the assessment scope [31]. Equally, full transparency on any conflict of interest as well as a reporting duty on stakeholder participation are anchored in the regulation (recital 29, articles 5 and 6.2 of the EU HTAR) [1]. It remains to be seen how the two principles of transparency and competency will be balanced in the upcoming procedures [27].

KPIs covering the Health System Goal ‘efficiency of the health system’:

A variety of suggested prioritized KPIs are related to the ‘efficiency of the health system’. At this early stage of implementing the EU HTA process, it is apparent that the attention is primarily focused on procedural KPIs measuring the system’s functioning, in addition to the regulation’s overarching goal of improving health systems. As such, recital 13 of the EU HTAR emphasizes the need to avoid duplication of efforts and increase efficiency [1]. This is further emphasized by article 31.1 (b) of the EU-HTAR, according to which the avoidance of duplication in requests for data and evidence is a focal point in the Commission’s report on the regulation’s application [1]. Respective KPIs were prioritized both from a systems’ reduction in workload as well as from an HTD workability, realistic response timelines, and efficient communication perspective. Furthermore, all four stakeholder groups indicated the relevance of a KPI that addresses the availability of beneficial medicines to patients in the various members states and the time to patient access. This corresponds with recital 36 of the EU HTAR and recital 26 of the JCA-IR, which highlight the improvement of timely patient access as objectives of the respective regulations [1,31]. Advice for HTDs in order to inform clinical development programs as implemented via JSCs is a critical factor enabling efficiency and is therefore anchored in the EU HTAR [1]. A KPI related to JSC was therefore HTD-prioritized and strongly supported by all four stakeholder groups. However, the currently prevailing perception is that the JSC and JCA instruments are not equally developed and that it is a major omission, particularly in this early phase of EU HTA, that the JSC component, which may support the generation of clinical evidence for high quality JCA submissions, is not appropriately developed. This perceived shortcoming might also contrast the importance attributed to scientific evidence for clinical decision-making and patient access as per recitals 2 and 5 of the EU HTAR [1].

KPIs covering the Health System Goal ‘equity of the health system’:

Finally, equity of patient access was prioritized as a KPI from a patient perspective, which aligns with the health system goal of ‘equity of the health system’. This corresponds with recitals 3, 8, 9, and 36 of the EU HTAR, which highlight the role of HTA and the HTAR framework in improving patient access [1]. However, irrespective of the overall goal of strengthening the ‘European Health Union’ [2], it must be acknowledged that this KPI depends on factors beyond EU HTA [36,37,38,39]. The setup of national health systems including processes and timelines for HTA and reimbursement decision-making, fragmentation, e.g., where regional authorities rather than centralized national authorities make those decisions, and budget constraints in national health care are important factors influencing patient access to innovative beneficial health technologies. With health systems and financial capabilities of the various EU member states differing considerably, this KPI would have a more aspirational, long-term perspective in order to capture bottlenecks and set expectations for aligned and timely processes across MS.

Limitations and further research

The presented list of KPIs prioritized by each of the relevant stakeholder groups involved in EU HTA represents a starting point for EU HTA performance measurement [34,40]. Adjustment of this prioritized KPI list, taking into account some of the components of the initially developed pool of KPIs (Table 1), might be required over time to reflect the evolving reality of the EU HTA process. Further, considering the overlap in focus topics for KPIs between stakeholders, segmentation of the identified KPIs in a matrix by EU HTA aspect covered might provide additional insights and validation of the framework in future work. Overall, the timely development of an inclusive multi-stakeholder performance measurement framework will provide a valuable source of input for the revision of the EU HTAR planned for 2028 to inform, complement, and support the efforts by the HTA Coordination Group and the European Commission [1]. In addition, it is an important opportunity for policymakers to secure health systems improvement and accountability on the way to a balanced European health system [1,18,35].

The development of details regarding the measurement of the identified KPIs, such as the metrics themselves, the measurement timepoints, etc., is a crucial part of EAA’s future work agenda. This might include matching the respective measurement approach to established methods such as the Balanced Scorecard introduced by Kaplan and Norton [41]. A key challenge in the future will be the development of baseline data for each of the suggested KPIs as a starting point for any measurement [42]. Due to the complexity and the need for thorough, in-depth evaluation of existing measurement projects, details regarding data sources, collection and analysis, baseline values, etc., this was beyond the scope of the current manuscript. At the EAA Fall Convention at Université Paris Cité in November 2025, a measurement framework based on the outcomes of this current work was discussed and it will be further developed for publication.

Empirical testing and validation of the developed KPI framework are still pending, as EU HTA procedures only began in 2026, with the first reports and further details not expected to become public until late Q1, 2026. In addition, the subsequent national processes in the Member States will take several weeks to months, meaning that initial data—such as on time to and equity of patient access—are unlikely to become available before late 2026. Validation, amendment, and optimization as in the envisioned learning system will therefore not be feasible until then. While in the mid- to long-term, ongoing development of quantitative data for each of the stakeholder groups is envisioned, and initially, qualitative assessments may prevail.

Finally, it is important to note that the presented performance measurement framework is restricted to performance components that are directly related to the implementation EU HTAR and performance of the joint procedures. However, there was broad agreement across all authors that the regulation should also have effects on the applied processes within each of the stakeholder groups. As such, the regulation is a unique opportunity for patients to strengthen their voice on European and national levels and to foster collaboration. For clinicians, the development of up-to-date living clinical guidelines that may (not only) inform the scoping and assessment process is a major challenge ahead. HTDs are charged with the development of convincing relative effectiveness data, preferably from randomized controlled trials. While measurement of those indirect effects of the regulation goes beyond the scope of this manuscript, they are an important element of a future research agenda. In addition, KPIs referring to equity of or time to patient access, which are clearly stated as aim of the EU HTAR and JCA-IR, would be influenced by other factors including health system characteristics, budgetary constraints, or delays in access and reimbursement requests by HTDs. Nevertheless, these KPIs were considered critical and necessary to include in any meaningful KPI framework for EU HTA [1,43,44,45]. On a system level, the relevance of the health system goals described by the WHO are found to be still relevant overall [19]; however, it will be important to consider a range of topics with increasing relevance, e.g., sustainability and resilience of health systems, integration of real-world evidence, payer willingness to pay, etc., in future research on HTA performance measurement.

This work aims to cover the perspectives of all EU member states; however, representation of stakeholders and geographical and national differences are limited despite the efforts to ensure a broad representation. Nevertheless, a range of stakeholder groups and national backgrounds were represented in the surveys (Delphi-Steps 1 and 2) and in the participants in the WG at the convention (Supplementary Figure S1). Geographical representation covered a variety of European countries and their unique national experiences within each stakeholder group. Overall, there was still a broad range of perspectives represented, resulting in diverse viewpoints being included in the outcomes.

## 5. Conclusions

To support the implementation of the EU HTAR, an inclusive multi-stakeholder framework for systematically measuring the performance and support of evidence-based optimization of the EU HTA system was developed. The identified stakeholder-centric KPIs, which align with the health system goals of the WHO, are the basis for the envisioned performance measurement. Future work will cover measurement details and metrics, i.e., what exactly to measure, when to measure, and the source of information or whom to approach for the required information. KPI measurement results are envisioned to provide evidence to inform the 2028 revision of the EU HTAR.

## Figures and Tables

**Table 1:** Delphi Approach—Steps 1–2: Stakeholder-centric KPIs as identified prior to the convention.

Outcomes Delphi-Step 1–2 (Pre-Convention)
Positive developments in health care system performance
European scope (PICO) reflecting the most clinically relevant population(s), comparator(s), and patient-relevant outcomes, that are manageable in size
Recognition of innovative/disruptive treatments
Stakeholder involvement and feedback (integration of input from right patient and clinical experts, etc.)
Utilization/adoption of JCA Assessment Report in national procedures and reduction in complementary data requests at member state level
Reduced time to patient access on national level
Earlier initiation of national decision-making processes and more transparent and predictable timelines
Reduced type I and type II errors (i.e., ensure that beneficial medicines are reaching patients)
Low number/low percentage of declined JSC requests
Inclusive/broad member state participation (e.g., as (co-)assessors)
Homogeneity and acceptance of state-of-the-art methodology across EU
JCA efficiency review (number of JCAs/time/costs/processes)
Equal/homogenous quality of patient access across EU
Equal access to innovative health technologies for patients in EU vs. US

Abbreviations: EU: European Union, JCA: Joint Clinical Assessment, JSC: Joint Scientific Consultation, KPI: Key Performance Indicator, MS: Member State, PICO: Patient/Intervention/Comparator, Outcome, US: United States.

**Table 2:** Summary of final prioritized stakeholder-centric KPIs (step 4) for EU HTA.

	Patient Perspective	Clinician Perspective	HTD Perspective	System-Level/MS Perspective
**Common KPIs**	- Time to patient access ^§^	- Time to patient access ^§^	- Shorter duration of the national decision-making process	- Time to patient access ^§^
	- Equity of patient access (scope: availability across MS) ^§^	- Equity of patient access (scope: availability across MS) ^§^		- Equity of patient access (scope: availability across MS contingent on budgetary capacities) ^§^
		- PICO-related KPI (scope: i) Reduction in the number of PICOs over time for a specific disease; and (ii) reflecting clinical standards in MS	- PICO related KPI (scope: optimization)	- PICO related KPI (scope: exhaustiveness); - Learning and training the system
	- Successful and meaningful patient involvement	- Successful and meaningful clinician involvement		
**Individual KPIs**	- Utilization of patient-centric/relevant outcome measures	- Addressing uncertainty (scope: clarity on strength/convincing outcomes)	- Functioning JSC process (scope: addressing existing demand and developmental timelines)	- Reduce duplication of effort (scope: utilization of JCA report and reduction in workload)
		- Transparency	- Functioning JCA process (scope: workability/realistic response timelines/efficient communication)	

^§^ influenced by other factors. Abbreviations: HTD: Health Technology Developer, JCA: Joint Clinical Assessment, JSC: Joint Scientific Consultation, KPI: Key Performance Indicator, MS: Member State, PICO: Patient/Intervention/Comparator/Outcomes, WG: Working Group, WHO: World Health Organisation.

## Data Availability

The raw data supporting the conclusions of this article will be made available by the authors on request.

## Supplementary Materials

The following supporting information can be downloaded at: https://www.mdpi.com/article/10.3390/jmahp14010005/s1, Figure S1: Distribution of stakeholder types in the break-out sessions.
