# Supplementary figures and images for: Development of a Performance Measurement Framework for European Health Technology Assessment: Stakeholder-Centric Key Performance Indicators Identified in a Delphi Approach by the European Access Academy
Source: J Mark Access Health Policy. 2026 Jan 15;14(1):5. doi: 10.3390/jmahp14010005 (PMC12821655; doi:10.3390/jmahp14010005)

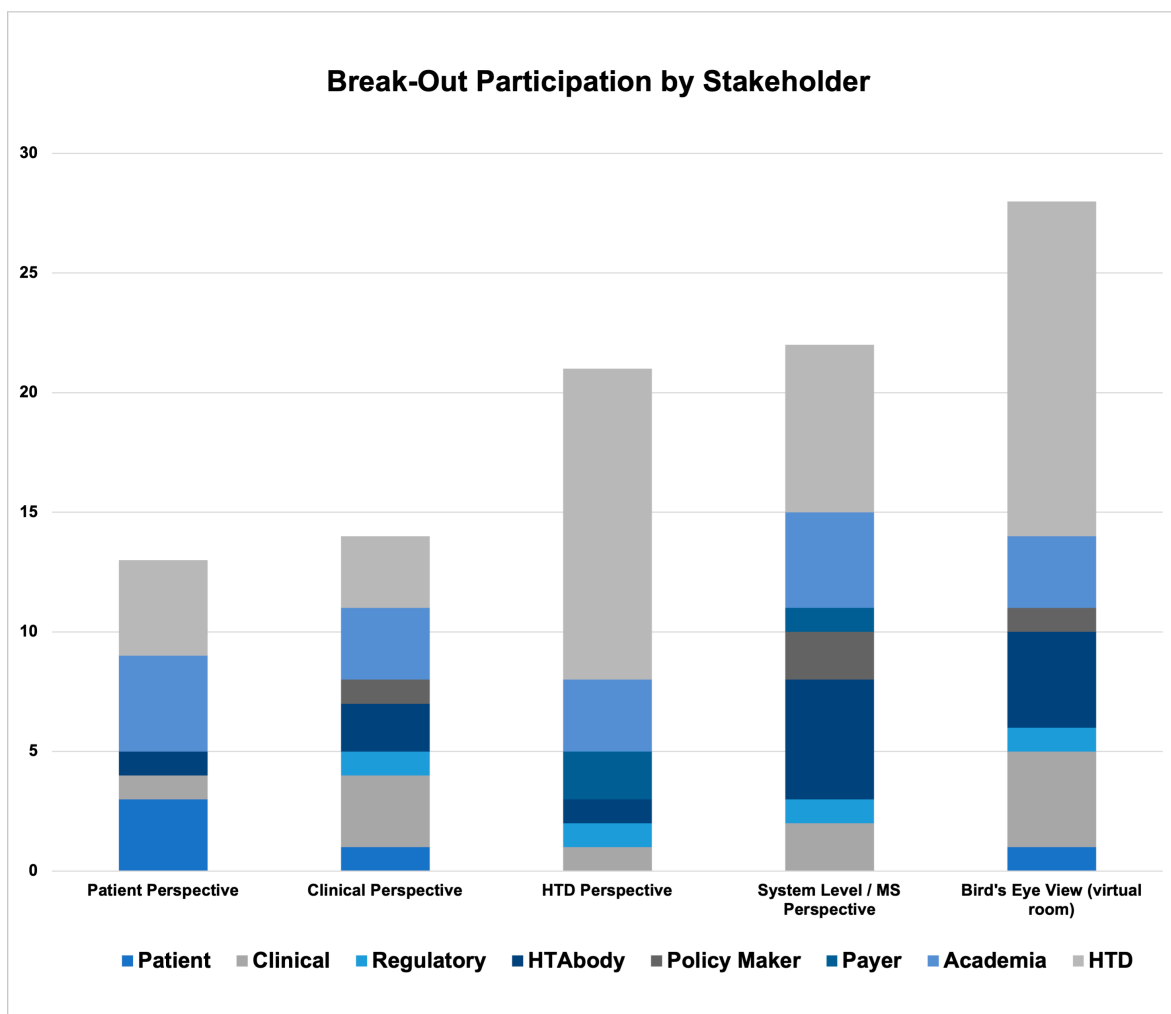

Figure S1. Distribution of stakeholder types in the break-out sessions.

Supplement: Supplementary file 1 [file jmahp-14-00005-s001.zip › jmahp-3809267-supplementary.pdf]
